# The Interconnection Between UbcH10, p53, and EGFR in Lung Cancer Cells and Their Involvement in Treatment Response

**DOI:** 10.3390/genes16040404

**Published:** 2025-03-30

**Authors:** Cristina Quintavalle, Umberto Malapelle, Marco De Martino, Danilo Rocco, Alfredo Fusco, Francesco Pepe, Claudio Bellevicine, Francesco Esposito, Pierlorenzo Pallante

**Affiliations:** 1 Institute of Endotypes in Oncology, Metabolism and Immunology (IEOMI) “G. Salvatore”, National Research Council (CNR), S. Pansini 5, 80131 Naples, Italy; cristina.quintavalle@cnr.it (C.Q.); francesco-esposito@cnr.it (F.E.); 2 Department of Public Health, University of Naples “Federico II”, S. Pansini 5, 80131 Naples, Italy; umberto.malapelle@unina.it (U.M.); francesco.pepe4@unina.it (F.P.); claudio.bellevicine@unina.it (C.B.); 3 Department of Molecular Medicine and Medical Biotechnology (DMMBM), University of Naples “Federico II”, S. Pansini 5, 80131 Naples, Italy; marco.demartino2@unina.it (M.D.M.); alfredo.fusco@unina.it (A.F.); 4 Department of Pulmonary Oncology, “V. Monaldi” Hospital, AORN Ospedali dei Colli, L. Bianchi, 80131 Naples, Italy; danilo.rocco@ospedalideicolli.it

**Keywords:** UbcH10, p53, EGFR, non-small cell lung cancer, adenocarcinoma, resistance

## Abstract

**Background/Objectives**: The UbcH10 protein plays an important role in a variety of human malignancies, including thyroid, breast, ovarian, and colorectal carcinomas. It has been previously reported that UbcH10 is overexpressed in non-small cell lung cancer (NSCLC) compared to normal lungs and that its expression is directly and inversely correlated with the mutational status of *p53* and *EGFR*, respectively. **Methods**: We transfected lung cancer cells with wild-type and mutant forms of *EGFR*, modulated the expression of UbcH10 and p53, and treated these cells with tyrosine kinase inhibitor (TKI) erlotinib. Using Western blotting, we evaluated the expression of UbcH10 induced by EGFR and p53. Finally, we employed immunohistochemistry to assess the levels of UbcH10 expression in a subset of NSCLC patients receiving TKI therapy. **Results**: We reported a possible modulation of UbcH10 expression by the overexpression of wild-type and mutant EGFR in H460 lung cancer cells, potentially through p53. The enforced expression of UbcH10 in cells transfected with mutant *EGFR* suggested a potential increase in resistance to erlotinib treatment. Finally, immunohistochemical analysis of samples from NSCLC patients with mutant *EGFR* indicated a possible connection between UbcH10 expression levels and progression-free survival. **Conclusions**: In NSCLC, UbcH10 may play a role in the regulation of TKI response via a molecular pathway potentially involving p53 and EGFR. However, further research is needed to fully understand this mechanism.

## 1. Introduction

Lung cancer is the major cause of cancer morbidity and mortality, accounting for 12.4% cancer diagnoses worldwide and 18.7% cancer deaths in 2022, with nearly 2.5 million new cases and over 1.8 million fatalities worldwide. Lung cancer incidence and fatality rates are approximately twice as high in males as in females, making the illness rank first in men and second in women. However, this scenario differs significantly by region, ranging from nearly equal in North America and Northern Europe to four to five times in Northern Africa and Eastern Europe [1].

Non-small cell lung cancer (NSCLC) and small cell lung cancer (SCLC) account for 85% and 15% of all lung cancers, respectively. More precisely, NSCLC is subdivided into lung adenocarcinoma (AD, 40% of all lung cancers), squamous cell carcinoma (30% of all lung cancers), large cell carcinoma (15% of all lung cancers), and other uncommon histotypes like adenosquamous and sarcomatoid carcinomas. Even if SCLC accounts for only around 15% of all lung malignancies, it presents management difficulties since it is more likely to grow and spread than NSCLC [2].

The clinical treatment of NSCLC has dramatically changed in the last decade due to an advanced molecularly customized strategy. Additionally, more complex and therapeutically useful subsets are made possible by including molecular classification in the conventional histological classification of lung cancer (NSCLC vs. SCLC). This paradigm shift emphasizes the advancements in personalized treatment for lung cancer and the role that molecular diagnostics plays in guiding therapeutic decision-making [3].

Identifying specific driver mutations in genes essential to proliferation and survival pathways, including *EGFR* and others like *ALK*, *ROS1*, and *KRAS*, in a subset of NSCLC has sparked the creation of more potent treatment approaches. By focusing on *EGFR* mutant variants and overcoming resistance mechanisms, third-generation EGFR inhibitors, such as osimertinib, have greatly improved patient outcomes in precision oncology [4,5].

First discovered in 2004, *EGFR* mutations are mostly found in lung AD and are significantly linked to better clinical outcomes than tumors expressing wild-type EGFR (EGFR-WT) [6,7]. Treatment with tyrosine kinase inhibitors (TKIs), such as osimertinib or erlotinib, frequently results in a survival period of more than two years for patients with metastatic *EGFR*-mutant cancers, demonstrating the effectiveness of targeted therapy [4,5]. Nonetheless, treatment resistance continues to be a major problem, appearing as either acquired resistance (brought on by bypass pathway activation) or secondary mutations [8,9]. A key focus in enhancing outcomes for these patients is addressing resistance mechanisms using combination treatments and next-generation inhibitors. This phenomenon restricts the effectiveness of therapeutic interventions, and translational research is heavily focusing on finding biomarkers that can predict the development of resistance. This, in fact, can increase the fraction of patients who show a significant therapeutic response to TKIs while simultaneously lowering the occurrence of adverse effects.

An essential enzyme in the ubiquitin–proteasome system, UbcH10 (encoded by the *UBE2C* gene), participates in the degradation of proteins that control the progression of the cell cycle. UbcH10 overexpression is linked to tumor growth and a poor prognosis and has been observed in a variety of human carcinomas, including those of the breast, lung, ovary, cervix, and colon [10,11,12,13]. Its overexpression may promote carcinogenesis by causing chromosomal instability and aneuploidy [14], and a poor prognosis has been linked to elevated UbcH10 protein expression in esophageal squamous cell carcinoma [15]. Furthermore, it has been demonstrated that the inhibition of UbcH10 expression increases susceptibility to chemotherapy and inhibits tumor cell proliferation, underlining its potential as a therapeutic target [16]. These results highlight the role that UbcH10 plays in the development and progression of human carcinomas and raise the possibility that it may be used as a target for therapeutic intervention as well as a biomarker for cancer prognosis establishment [13].

Interestingly, UbcH10 expression is inversely correlated with the presence of mutations in the *EGFR* tyrosine kinase domain, as it has been previously reported [17]. This can argue, at least in principle, that UbcH10 may also serve as a biomarker for the dynamic assessment of resistance to TKI treatment in NSCLC. The purpose of this work, therefore, was to investigate whether different UbcH10 levels are linked to resistance to TKI treatment and to determine why UbcH10 levels are lower in NSCLC with *EGFR* mutations compared with *EGFR*-WT tumors. We discovered that EGFR variants affect UbcH10 expression through a mechanism that also involves p53 modulation, and that increased UbcH10 expression levels could be associated with the appearance of TKI resistance in lung AD patients.

## 2. Materials and Methods

### 2.1. Cell Cultures and Transfections

The lung cancer cell lines H460, A549, and Calu-1 were employed in this investigation. They were kept at 37 °C in a humidified atmosphere with 5% CO_2_ (to optimize the pH of the culture medium), with the balance being ambient air. A549 and Calu-1 cells were grown in DMEM, while H460 cells were grown in RPMI 1640 (Gibco Laboratories, Carlsbad, CA, USA), and both media were supplemented with ampicillin/streptomycin, glutamine, and 10% fetal bovine serum.

Following the manufacturer’s instructions, Lipofectamine^®^ 2000 Transfection Reagent (Invitrogen, Carlsbad, CA, USA) was used to perform transfections. Expression vectors containing human cDNA sequences corresponding to *EGFR*-WT, *EGFR*-L858R, and *EGFR*-T790M (from William Pao’s laboratory at Vanderbilt University), UbcH10, *p53*-WT, and *p53*-V143A were used to transfect lung cancer cells. The PcDNA 3.1 vector was used as the backbone negative control to normalize the amount of transfected DNA. RNA interference was used to silence the expression of p53 and UbcH10. SiRNA oligonucleotides were acquired from Qiagen (Qiagen, Valencia, CA, USA). As a transfection and negative control, a fluorescent non-silencing control (cat. n. 1022563, Qiagen) was used. After performing pilot experiments to ensure the best achievable conditions in terms of non-toxicity, target specificity, and extent of silencing, the siRNA oligonucleotides were transfected at a final dose of 120 nM in a 60 mm dish plated with 0.5 × 10^6^ cells.

### 2.2. Proliferative Studies

Viability was assessed using Cell Proliferation Kit I (MTT, Roche Diagnostics, Mannhein, Germany) in the 96-well format. In total, 7 × 10^3^ cells were plated in each well, and 24 h after treatment, the absorbance was measured at 550 nm–690 nm using an Absorbance Microplate Reader ELx800 (BioTek Instruments, Inc., Winooski, VT, USA). The results achieved were compared to the control group that did not receive treatment. Erlotinib was purchased from Cayman Chemical (#10483, Cayman Chemical, Ann Arbor, MI, USA) and was administered to the cells 24 h after transfection.

Apoptosis was evaluated using a Promega Caspase-Glo™ 3/7 Assay Kit (Promega Corporation, Madison, WI, USA) following the manufacturer’s instructions.

### 2.3. Protein Extraction, Western Blotting, and Immunoprecipitation

The protocols for protein extraction and Western blotting were followed as previously described [18]. Following SDS-PAGE and blotting, membranes were incubated with primary antibodies that recognized EGFR (Santa Cruz Biotechnology, Inc., Dallas, TX, USA), p53 (Santa Cruz Biotechnology, Inc.), UbcH10 (A-650, Boston Biochem Inc., Cambridge, MA, USA), and β-Actin (Clone AC-15 A5441, Sigma-Aldrich Co., St. Louis, MO, USA). After primary antibody incubation, the membranes were incubated with the HRP-conjugated secondary antibody (dilution of 1:3000) for 60 min at room temperature. Finally, the reaction was revealed using a chemiluminescent Western blotting detection kit (Pierce™ ECL Western blotting Substrate, Thermo Fisher Scientific, Waltham, MA, USA). Finally, films were developed using a Cawomat IR 2000 developer (CAWO Photochemisches, Schrobenhausen, Germany).

Immunoprecipitation analysis was performed as reported elsewhere [18] by employing anti-ubiquitin (Boston Biochem Inc.) and anti-p53 (Santa Cruz Biotechnology, Inc.) antibodies, where appropriate. HEK 293 cells were used for transfection and interaction analysis.

### 2.4. Selection of NSCLC Tissue Specimens

NSCLC tissue specimens were stained and analyzed at the University “Federico II” Hospital (Naples, Italy). The selected patients had lung AD harboring activating mutations in the *EGFR* gene (exons 19 and 21) and had not received any prior chemotherapy or radiation treatment. The patients were treated with gefitinib/erlotinib upon the discovery of *EGFR* mutations during the follow-up [19], and the disease’s course was assessed at various points throughout treatment. Two-dimensional measures of the gadolinium-enhancing mass on MRI or CT (if MRI was not recommended) at eight-week intervals were used to assess the patients’ response to the treatment. The responses that were observed were as follows: stable disease (SD, no change in mass size); partial response (PR, not complete reduction of enhancing mass); complete response (CR, complete absence of gadolinium-enhancing mass); mixed response (MR, variable change of multiple masses including progression with concomitant PR or CR); and progressing disease (PD, increased diameter of enhancing gadolinium mass). Two expert pathologists (UM and CB) examined the slides and evaluated the UbcH10 staining at the time of diagnosis, before beginning treatment.

Based on these criteria, in this analysis, we included 11 cases (n = 11) of lung AD with activating mutations in the tyrosine kinase domain of *EGFR*, and three cases (n = 3) of NSCLC carrying *EGFR*-WT.

### 2.5. Immunohistochemistry

Lung AD slides were stained by applying immunohistochemical protocols, as described elsewhere [17]. To reveal antigens, slides were briefly placed in a pressure cooker for three minutes following deparafinization and rehydration. The slides were incubated overnight with anti-UbcH10 antibody (Boston Biochem Inc.) and anti-rabbit secondary antibody. Finally, a DAB substrate was added, and UbcH10 signals were then revealed. Negative control slides were also included in the analysis by omitting the main antibody. Two pathologists (UM and CB) scored the slides by evaluating at least 500 cells in each field using a semi-quantitative grading method that considered both the UbcH10-expressing cell percentage and signal intensity (strong, 3+; moderate, 2+; weak, 1+). The simplified H-score was calculated by multiplying the staining intensity value by the percentage of positive cells.

### 2.6. Statistical Methods

Where appropriate, two-way ANOVA, followed by Bonferroni post-tests, was used for the statistical analyses. For every analysis, *p*-values less than 0.05 were considered statistically significant and indicated in the text; non-significant *p*-values were not reported in the text. GraphPad Prism software (version 5.0) was used to make statistics and generate graphs.

## 3. Results

### 3.1. UbcH10 Modulates Drug Sensitivity of Lung Cancer Cells Carrying Mutant EGFR

**Figure 1:**
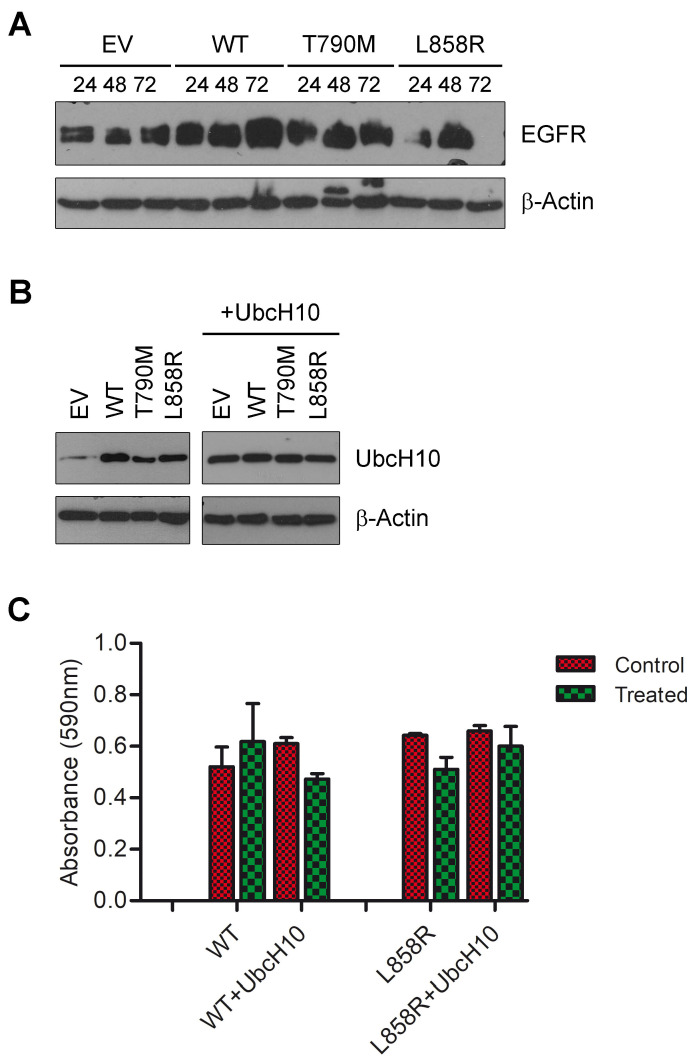
UbcH10 modulates the drug sensitivity of EGFR-L858R H460 cells to erlotinib treatment. (**A**) The empty vector (EV) and the three *EGFR* constructs (WT, T790M, and L858R) were transfected into H460 cells, and the expression of the EGFR protein was checked at three different time points (24, 48, and 72 h after transfection). β-Actin was assessed as a normalization control. Representative images. (**B**) Levels of UbcH10 protein were evaluated using Western blotting after transfection of *EGFR*-WT, *EGFR*-T790M, and *EGFR*-L858R in H460 cells (**left** blot). The enforced expression of UbcH10 following co-transfection of *EGFR*-WT, *EGFR*-T790M, *EGFR*-L858R, and *UbcH10* in H460 cells (**right** blot) was also evaluated. The **left** and **right** blots are from two different Western blots. β-Actin was evaluated as a normalization control. Representative images. EV, empty vector. (**C**) H460 cells were treated with 0.1 μM erlotinib after transfection of *EGFR*-WT and *EGFR*-L858R, and their viability was assessed 24 h after treatment (**left**). H460 cells were also treated with 0.1 μM erlotinib after co-transfection of *EGFR*-WT, *EGFR*-L858R, and *UbcH10*, and their viability was assessed 24 h after treatment (**right**). Data are in triplicate and are shown as mean ± sd. Two-way ANOVA: treatment, *p* = 0.005.

Once we discovered that the overexpression of UbcH10 was inversely correlated with the presence of *EGFR* mutations in NSCLC specimens [17], we asked whether UbcH10 expression could be involved in the modulation of sensitivity to therapy. Therefore, we transfected H460 lung cells with vectors expressing *EGFR*-WT and two mutant forms, *EGFR*-L858R and *EGFR*-T790M (Figure 1A). PcDNA 3.1 empty vector was used as a negative control. After assessing the UbcH10 expression levels, we found that whereas transfection with the *EGFR* mutant forms resulted in only a moderate overexpression of UbcH10, transfection of *EGFR*-WT in H460 cells induced an intense overexpression of the UbcH10 protein (Figure 1B, left). We used H460 cells, since they express low basal levels of UbcH10, as previously reported [17]. These results appear to support our previous findings, suggesting that different EGFR forms (wild-type and mutants) may differentially induce UbcH10 overexpression. Specifically, there seems to be a trend where lower UbcH10 levels are associated with *EGFR* mutations [17].

Subsequently, we treated *EGFR*-WT and *EGFR*-L858R H460-transfected cells with erlotinib and measured their ability to survive. Although cells expressing EGFR-WT were insensitive to treatment (EGFR-WT treated vs. EGFR-WT, 118.8% of viability; Figure 1C, left), cells expressing EGFR-L858R were sensitized to this treatment (EGFR-L858R treated vs. EGFR-L858R, 79.4% of viability; Figure 1C, left). This indicates that the EGFR-WT and EGFR-L858R conferred different responsiveness to erlotinib, confirming what is observed in patients in vivo.

Next, to assess any role of UbcH10 in this mechanism, we transfected H460 cells with the *EGFR* different forms together with a vector constitutively expressing the UbcH10 protein (Figure 1B, right), and treated cells carrying *EGFR*-WT and *EGFR*-L858R with erlotinib 24 h after transfection. We assessed cell viability 24 h after erlotinib treatment, and as reported in Figure 1C (right), the enforced expression of UbcH10 enhanced the viability of H460 EGFR-L858R cells (EGFR-L858R+UbcH10 treated vs. EGFR-L858R+UbcH10, 91.1% of viability), thereby making them a more resistant phenotype; however, unexpectedly, the overexpression of UbcH10 in the EGFR-WT cells seems to enhance their sensitivity to treatment (EGFR-WT+UbcH10 treated vs. EGFR-WT+UbcH10, 77.4% of viability; Figure 1C, right). In conclusion, these results may indicate that NSCLC cells with EGFR mutations are potentially more resistant to TKI therapy when UbcH10 protein levels are higher.

### 3.2. Overexpression of UbcH10 Is Associated with Resistance in NSCLC Patients

**Figure 2:**
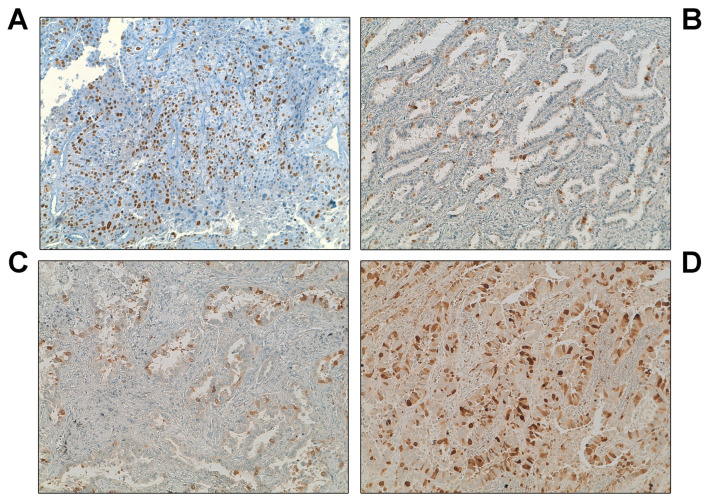
Higher levels of UbcH10 are associated with short progression-free survival in NSCLC patients. At the time of diagnosis, UbcH10 immunohistochemical expression and *EGFR* mutational status were assessed. Objective response was evaluated according to RECIST criteria with the first evaluation performed 8 weeks after the start of the treatment. The response rate was linked to UbcH10 levels at diagnosis. (**A**) Exon 19 and 21 wild-type patient (W1) characterized by high UbcH10 expression levels. (**B**) *EGFR* mutant patient (M5) showing low UbcH10 expression levels and a complete response to treatment. (**C**) *EGFR* mutant patient (M9) showing intermediate UbcH10 expression levels and stable disease. (**D**) *EGFR* mutant patient (M3) showing moderate to high UbcH10 expression levels and partial response.

To verify the association between *EGFR* mutations and reduced UbcH10 levels in in vivo specimens, we assessed the expression of UbcH10 in several NSCLC tissues derived from patients receiving treatment. N = 3 NSCLC specimens with *EGFR*-WT and n = 11 AD specimens with L858R or del746–750 mutations were examined (Table 1). According to the immunohistochemical analysis, AD specimens carrying the L858R or del746–750 mutations (Table 1, Figure 2B–D) showed lower UbcH10 expression levels compared with *EGFR*-WT specimens (Table 1, Figure 2A).

Since patients with these mutations become resistant to treatment after a very specific time, we looked at the progression-free survival (PFS) time of patients with *EGFR* mutations (Table 1) and related it to UbcH10 expression levels. Notably, patients who experienced extended treatment benefit (CR, H-score = 12.5; SD, H-score = 45) had lower UbcH10 levels, as determined by IHC, while those developing resistance quickly (PR, H-score = 61.7; PD, H-score = 45) showed higher levels (Table 1, Figure 2B–D). These results suggest a potential association between a higher rate of resistance development and elevated UbcH10 expression in patients carrying L858R or del746–750 sensitivity mutations. However, further investigation is warranted to confirm this observation.

### 3.3. P53 May Influence UbcH10 Expression in Lung Cancer Cells

**Figure 3:**
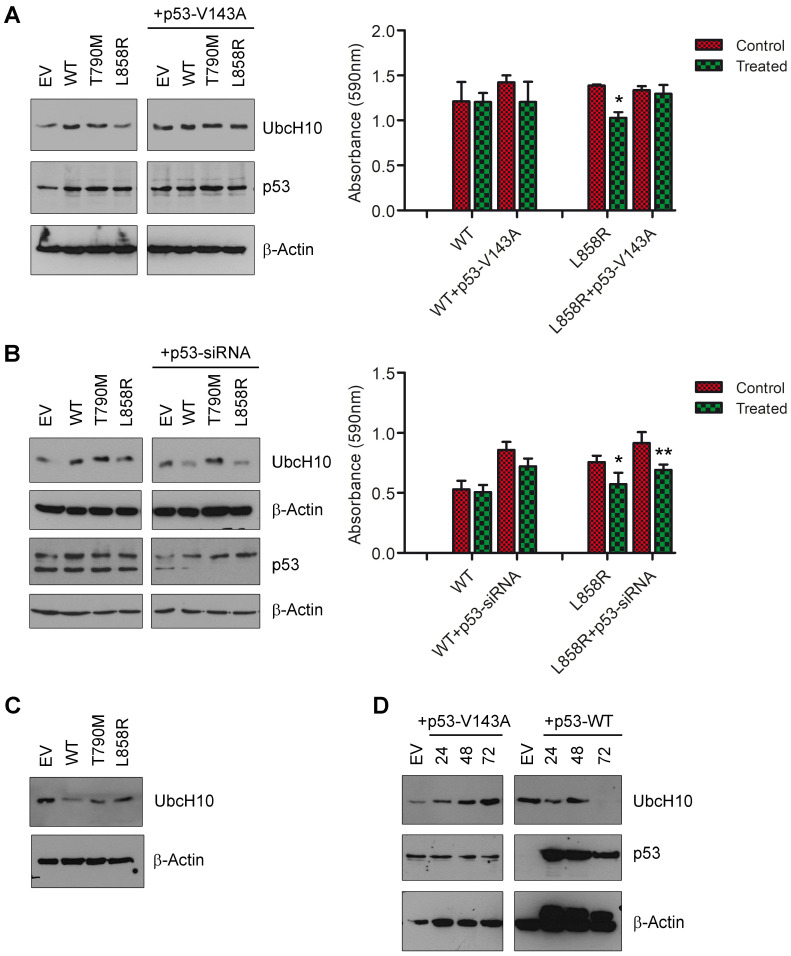
The expression of UbcH10 in lung cancer cells is under the control of p53. (**A**) **Left** panel. Levels of UbcH10 and p53 protein were evaluated after transfection of H460 cells with *EGFR*-WT, *EGFR*-T790M, and *EGFR*-L858R. UbcH10 protein expression was also evaluated after co-transfection of H460 cells with *EGFR* different forms and a vector expressing p53-V143A. β-Actin was evaluated to normalize the amount of protein loaded. Representative images. EV, empty vector. **Right** panel. Viability of H460 cells co-transfected with *EGFR*-WT, *EGFR*-L858R, and *p53*-V143A was evaluated 24 h after treatment with 0.1 μM erlotinib. Data are in triplicate and are shown as mean ± sd. Two-way ANOVA: EGFR-L858R treated vs. EGFR-L858R, * *p* < 0.05. (**B**) **Left** panel. Levels of UbcH10 and p53 (two different Western blots) were evaluated after the co-transfection of *EGFR*-WT, *EGFR*-T790M, and *EGFR*-L858R with siRNA suppressing the p53 expression. UbcH10 levels were evaluated 48 h after silencing p53 expression. β-Actin was evaluated to normalize the amount of protein loaded. Representative images. EV, empty vector. **Right** panel. Viability of H460 cells co-transfected with *EGFR*-WT, *EGFR*-L858R, and *p53*-siRNA was evaluated 24 h after treatment with 0.1 μM erlotinib. Data are in triplicate and are shown as mean ± sd. Two-way ANOVA: treatment, *p* = 0.0021; +p53-siRNA, *p* < 0.0001; EGFR-L858R treated vs. EGFR-L858R, * *p* < 0.05; EGFR-L858R+p53-siRNA treated vs. EGFR-L858R+p53-siRNA, ** *p* < 0.01. (**C**) Levels of UbcH10 protein were evaluated after transfection of Calu-1 cells with *EGFR*-WT, *EGFR*-T790M, and *EGFR*-L858R. β-Actin was evaluated as a normalization control. EV, empty vector. (**D**) UbcH10 protein expression was evaluated at 24, 48, and 72 h after transfecting *p53*-V143A and *p53*-WT in H460 (**left** blot) and Calu-1 (**right** blot) cells, respectively. β-Actin was assessed as a control for normalization. EV, empty vector.

In an effort to understand the modulation of UbcH10 expression after transfecting H460 cells with different *EGFR* forms, we observed that cells expressing EGFR-WT and mutants had higher p53 protein levels compared to the same cells transfected with the empty vector (Figure 3A, left panel). This finding led us to believe that there might be a connection between these two events. Consequently, in order to confirm any role of p53 in the modulation of UbcH10 expression, we carried out *EGFR* transfection following the silencing of p53. As shown in Figure 3B, the expression of EGFR-WT upon p53 silencing was no longer able to induce a significant overexpression of UbcH10, as can be particularly observed in EGFR-L858R H460 cells (Figure 3B, left panel). These data provide evidence for a connection between EGFR and UbcH10 expression and suggest that a p53-related pathway may play a role in this process. In terms of treatment resistance, we found that following p53 silencing in L858R H460 cells, erlotinib treatment had the same effect (viability: EGFR-WT treated vs. EGFR-WT, 95.4%; EGFR-WT+p53-siRNA treated vs. EGFR-WT+p53-siRNA, 84%; EGFR-L858R treated vs. EGFR-L858R, 75.7%, two-way ANOVA, *p* < 0.05; EGFR-L858R+p53-siRNA treated vs. EGFR-L858R+p53-siRNA, 75.5%, two-way ANOVA, *p* < 0.01; Figure 3B, right panel), and this observation further associates treatment sensitivity with UbcH10 levels (Figure 3B, left panel).

Subsequently, since p53 was required for UbcH10 induction, we asked whether a mutant form of p53 could similarly impact the expression levels of UbcH10. Thus, we transfected the H460 cells (which carry *p53*-WT and express a small amount of UbcH10) with the *p53*-V143A mutant. It is interesting to note that the expression of p53-V143A in these cells could significantly induce the expression of the UbcH10 protein (Figure 3A, left panel, right blot). This result is in line with our previously published findings, which showed a positive correlation between UbcH10 expression and the presence of *p53* mutations in NSCLC patients [17]. Again, we treated these cells with erlotinib and observed that in the presence of p53-V143A, the H460-L858R cells became resistant to treatment (viability: EGFR-WT treated vs. EGFR-WT, 99.4%; EGFR-WT+p53-V143A treated vs. EGFR-WT+p53-V143A, 84.8%; EGFR-L858R treated vs. EGFR-L858R, 74%, two-way ANOVA, *p* < 0.05; EGFR-L858R+p53-V143A treated vs. EGFR-L858R+p53-V143A, 97.1%; Figure 3A, right panel). This result appears to align with the overexpression of UbcH10 induced by the p53 mutation and suggests that the observed resistance to treatment could be associated with increased UbcH10 levels (Figure 3A, left panel, right blot). However, further studies are needed to confirm this connection.

Nevertheless, since it seems that p53 is crucial for the expression of UbcH10 in both the wild-type and mutant versions, we tried to further clarify this point. We investigated the effects of transfection of *EGFR*-WT and mutants in lung cells that do not express p53 (Calu-1, p53-deleted) but express high amounts of UbcH10. After transfection, the levels of UbcH10 in these cells were assessed using Western blotting. We found that *EGFR*-WT transfection significantly reduced the expression levels of UbcH10. On the other hand, UbcH10 expression levels were weakly affected by the transfection of mutant *EGFR* forms (Figure 3C).

Finally, to verify the role of p53 in the downregulation of UbcH10 caused by EGFR in Calu-1 cells, we transfected these cells with *p53*-WT and H460 cells with *p53*-V143A, and evaluated the levels of UbcH10 at 24, 48, and 72 h. Interestingly, we observed that while p53-V143A was able to induce UbcH10 in H460 cells (Figure 3D, left blot), the enforced expression of p53-WT in Calu-1 cells was able to strongly reduce the expression of the UbcH10 protein (Figure 3D, right blot). This appears to provide a potential mechanism for how EGFR acts on UbcH10 through p53, indicating a possible role for p53. However, further investigation is required to achieve comprehensive validation of the proposed model.

### 3.4. Potential Self-Regulatory Relationship Between UbcH10 and p53

**Figure 4:**
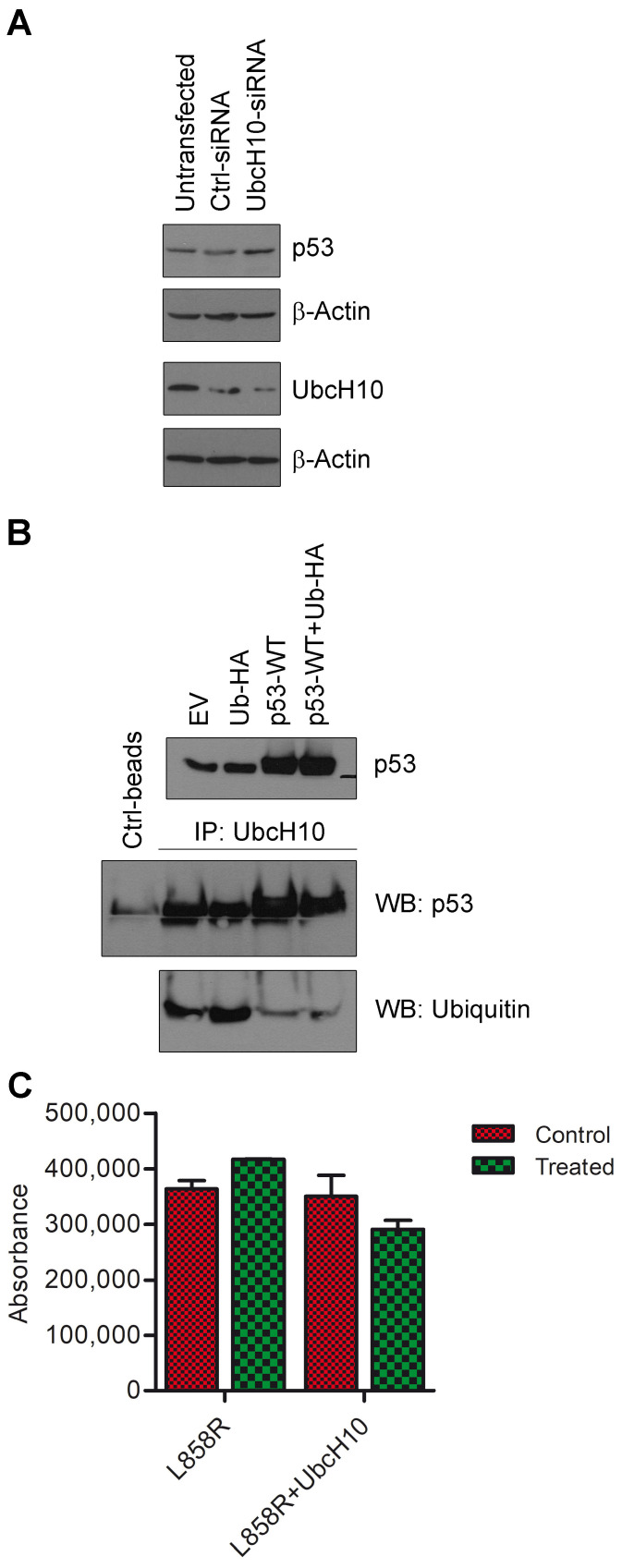
UbcH10 and p53 are part of a self-regulatory mechanism. (**A**) P53 and UbcH10 expression levels (two different Western blots) were evaluated 48 h after transfection of A549 cells with siRNA able to reduce the expression of UbcH10. β-Actin was evaluated as a normalization control. (**B**) HEK 293 cells were transfected with empty vector, *ubiquitin*-HA, *p53*-WT, and *p53*-WT+*ubiquitin*-HA, and p53 protein expression levels were evaluated. Protein lysates were immunoprecipitated with anti-UbcH10 antibodies, and levels of UbcH10/ubiquitin complexes were evaluated using Western blotting with an anti-ubiquitin antibody. EV, empty vector. (**C**) H460 cells were co-transfected with *EGFR*-L858R and *UbcH10*. Caspase activity was evaluated using caspase assay 24 h after treatment with 0.1 μM erlotinib. Data are in duplicate and are shown as mean ± sd. Two-way ANOVA: interaction, *p* = 0.0303; +UbcH10, *p* = 0.0201.

After it was determined that p53 plays a role in regulating UbcH10 expression, we tried to investigate the mechanism behind this phenomenon. After excluding the possibility that UbcH10 was under the transcriptional control of EGFR, we evaluated the possibility that UbcH10 and p53 could directly interact and reciprocally regulate themselves. Interestingly, it has already been demonstrated that p53 is indeed a target of UbcH10 [20,21,22,23], and previous works, aimed at clarifying the mechanism by which UbcH10 regulates the cell cycle, revealed a self-ubiquitination mechanism that triggers UbcH10 self-degradation after the complete depletion of its target proteins, Cyclin A and B [24]: thus, in our proposed mechanism, p53 may act as a target of UbcH10, allowing its self-regulation. To demonstrate this, we transfected A549 lung cancer cells with siRNA directed against UbcH10 [25], and as we can observe in Figure 4A, after UbcH10 reduction, the levels of p53 protein increased (Figure 4A). Subsequently, we used the anti-UbcH10 antibody to immunoprecipitate total protein from HEK 293 cells transfected with *p53*-WT and a vector expressing the ubiquitin-HA protein. Following SDS-PAGE, we performed a Western blotting by employing the anti-p53 antibody and observed that UbcH10 and p53 interact, as confirmed by the blot against p53 after immunoprecipitation with anti-UbcH10 antibody (Figure 4B). Subsequently, we employed an anti-ubiquitin antibody, and as illustrated in Figure 4B, we found that following p53 overexpression, the levels of ubiquitinated UbcH10 decreased. This suggests that the ability of UbcH10 to ubiquitinate itself was suppressed (by the abundant expression of p53), leading to its accumulation.

To further explore the significance of this molecular axis involving p53 and UbcH10, we also assessed the potential influence of UbcH10 on the apoptosis process. To determine whether UbcH10 may be involved in the modulation of apoptosis during erlotinib treatment, we assessed apoptosis through caspase assay in H460 cells transfected with *EGFR*-L858R, either alone or in combination with UbcH10 (Figure 4C). As shown in Figure 4C, the overexpression of UbcH10 upon EGFR-L858R expression decreased caspase activity after treatment, conferring a more resistant phenotype to the treated cells (viability: EGFR-L858R treated vs. EGFR-L858R, 114.6%; EGFR-L858R+UbcH10 treated vs. EGFR-L858R+UbcH10, 83%; Figure 4C). This preliminary observation appears to support the idea that UbcH10 plays a role in treatment resistance, potentially in conjunction with EGFR and p53.

## 4. Discussion

Since it was previously demonstrated that mutations in the *EGFR* gene correlated with a low expression of UbcH10 [17], we tried to understand if there was a relationship between UbcH10 and EGFR at the molecular level. Interestingly, we observed that the expression of different forms of EGFR (wild-type and mutant) was able to modulate UbcH10 expression differently. This observation immediately led us to believe that UbcH10 could have a role in modulating the response to TKI treatment, since the mutant EGFR forms induced only a low expression of UbcH10 compared with the wild-type form, which instead induced a strong overexpression.

We then investigated whether this mechanism was also present in vivo and not only in cell cultures. Therefore, we evaluated the expression of UbcH10 in a group of patients affected by lung AD who, at the time of diagnosis and assessment of *EGFR* mutations, started treatment with TKI. We confirmed that the expression of UbcH10 in AD carrying *EGFR* mutations tends to be lower, nevertheless showing a degree of variability. Then, we examined whether the expression of UbcH10 at this stage might be related to the PFS time. Specifically, we noted that lower UbcH10 levels at the beginning of therapy showed a trend towards a longer PFS, and higher levels towards a shorter PFS. Therefore, we propose to use the flexibility of UbcH10 in the diagnosis and treatment of NSCLC in fact, while the higher expression levels of UbcH10 might represent an early marker of developing resistance in patients treated with TKI, at the same time, the lower expression of UbcH10 could likely indicate the presence of *EGFR* mutations. We suggest that the analysis of UbcH10 may potentially improve patient selection for treatment, potentially increasing the response rate and reducing unnecessary care.

More intriguingly, we have found an interesting network involving EGFR, p53, and UbcH10, which may partly explain how EGFR modulates the expression of UbcH10 and how this latter could modulate resistance. Basically, at a molecular level, we found that EGFR can modulate UbcH10 expression via p53; in fact, the enforced expression of the different forms of EGFR induces first p53 and then UbcH10, since the silencing of p53 undermines this mechanism. Interestingly, transfection of a mutant form of *p53* can induce strong UbcH10 overexpression regardless of EGFR signaling. This observation could have interesting implications in vivo, as if in cells with *EGFR* mutations (sensitive to treatment) also occurs a mutation in *p53*, the levels of UbcH10 would tend to increase like that observed when *EGFR* is in the wild-type form. The positive correlation between UbcH10 levels and *p53* mutations, previously reported [17] and now corroborated at the molecular level, might be related to the potential development of treatment resistance in lung cancer patients.

**Figure 5:**
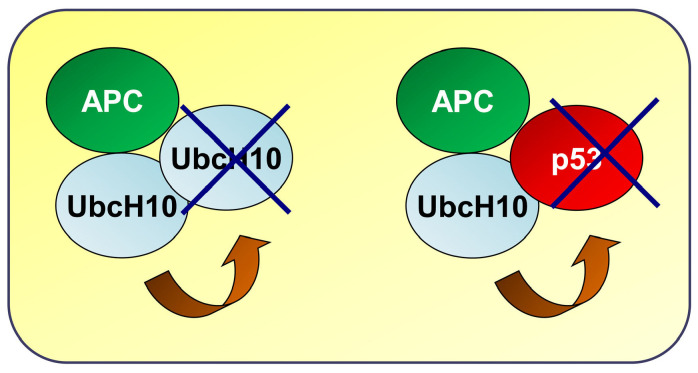
EGFR-p53-UbcH10 loop in the modulation of NSCLC cells to treatment. Schematic model likely showing UbcH10 overexpression in the presence of p53. In the absence of p53 target molecules, UbcH10 self-degrades, preventing accumulation. When p53 is present, UbcH10 targets it for degradation via the ubiquitin–proteasome system, resulting in UbcH10 accumulation and the activation of its oncogenic potential. APC, anaphase-promoting complex.

In this intriguing regulatory mechanism, however, it must also be considered that UbcH10 itself can target p53 for degradation, modulating its levels in the cell, as we observed in the present study and as also reported elsewhere [20,21,22,23]. Furthermore, as already reported for Cyclin A and B [24], the abundant presence of p53 could prevent UbcH10 from self-ubiquitination through the anaphase-promoting complex (APC), allowing its accumulation in the cell (Figure 5). This double-regulated mechanism, in turn, would change the balance between UbcH10 and p53 levels, disrupting cellular homeostasis that culminates with the acquisition of resistance.

As widely reported, p53 is also a master regulator of apoptosis [26] and its deregulation down to UbcH10 may affect the activation of the apoptotic process in lung carcinoma cells after treatment with TKI. Therefore, it is possible to hypothesize that while EGFR and its mutant variants stimulate p53, as a defense mechanism against oncogenic activation, p53 overexpression in turn stabilizes the UbcH10 protein (as a secondary effect), which may then carry out its oncogenic program. In light of this particular regulation, therefore, it might be even more critical to discriminate lung carcinomas based on the mutational status of *EGFR* and *p53*, together with UbcH10 expression levels. Specifically, elevated UbcH10 levels might be related to high p53 expression levels, which are known to increase when *p53* is altered [27].

Obviously, this work has several limitations, and some observations are very preliminary. For example, the case study analyzed in vivo is too small, and some molecular observations need to be investigated more thoroughly. This study reported a reduced treatment susceptibility when UbcH10 is overexpressed in the presence of the EGFR-L858R mutation, but the variable response in EGFR-WT cells following UbcH10 expression remains unexplained. The impact of UbcH10 on EGFR-WT cells suggests potential signaling disruption, yet the precise mechanism is unclear. In addition, this study does not provide a clear explanation of why different EGFR mutants affect UbcH10 in a different way. Additionally, p53 analysis was limited to one mutant isoform, and further research is needed on other mutations. In fact, cell models showed different degrees of p53 and UbcH10 expression, leading to diverse modulation of this latter. Finally, a significant challenge lies in understanding the counterintuitive activation of the oncogenic protein UbcH10 by the tumor suppressor p53. This phenomenon demands in-depth research, as its rationale is presently unknown. However, we believe that the proposed mechanism may have interesting future implications, especially in clinical practice, and for this reason, we propose its consideration.

## 5. Conclusions

In conclusion, this work indicates a potential mechanism in lung AD with *EGFR* mutations, where p53 and UbcH10 may influence resistance through an auto-regulatory system in certain patient subgroups.

## Figures and Tables

**Table 1:** Association of UbcH10 expression with response to TKI treatment.

Sample ^1^	Histotype ^1^	UbcH10 (% of Cells)	UbcH10 (Intensity)	Best Response ^2^	H-Score ^3^	H-Score (Mean)
W1	AD	60%	3+	n.a.	180	96.7
W2	SCC	10%	1+	n.a.	10	
W3	NSCLC-n.o.s.	50%	2+	n.a.	100	
M2	AD	5%	2+	CR	10	12.5
M5	AD	5%	3+	CR	15	
M4	AD	15%	2+	SD	30	45
M9	AD	30%	3+	SD	90	
M11	AD	5%	3+	SD	15	
M1	AD	10%	2+	PR	20	61.7
M3	AD	40%	3+	PR	120	
M8	AD	15%	3+	PR	45	
M10	AD	15%	3+	PD	45	45
M6	AD	5%	3+	n.e.	15	22.5
M7	AD	10%	3+	n.e.	30	

^1^ AD, adenocarcinoma; SCC, squamous cell carcinoma; NSCLC-n.o.s., non-small cell lung cancer-non otherwise specified; W1–W3, *EGFR* wild-type samples; M1-M11, *EGFR* mutated samples. ^2^ The response rate has been correlated to UbcH10 levels at diagnosis. Eight weeks following the initiation of treatment, the objective response was assessed using the RECIST criteria. CR, complete response; SD, stable disease; PR, partial response; PD, progressing disease; n.a., not applicable; n.e., not evaluable. ^3^ The simplified H-score was calculated by multiplying the staining intensity value by the percentage of positive cells.

## Data Availability

All data generated in the study are presented in this article. Further requests can be addressed to the corresponding author.

## Abbreviations

The following abbreviations are used in this manuscript:

NSCLC	Non-small cell lung cancer
SCLC	Small cell lung cancer
AD	Adenocarcinoma
TKI	Tyrosine kinase inhibitor
SD	Stable disease
PR	Partial response
CR	Complete response
PD	Progressing disease
APC	Anaphase-promoting complex
